# Bees as Ambassadors for Plant Awareness and Conservation

**DOI:** 10.1002/pei3.70171

**Published:** 2026-06-10

**Authors:** Ari E. Hoffman, Thais Vasconcelos, Eric R. Hagen

**Affiliations:** ^1^ Department of Ecology and Evolutionary Biology University of Michigan Ann Arbor Michigan USA

**Keywords:** bees, conservation, plant awareness, plant awareness disparity, plant blindness

## Abstract

Due to “Plant Awareness Disparity” (PAD) people tend to be unaware of, uninformed, or uninterested in the plants around them. Because this phenomenon contributes to a lack of support for the conservation of plants relative to animals, public awareness campaigns against PAD must be launched. Since the severity of PAD varies across demographic groups (e.g., gender, education, and age), such a campaign should be designed with demographic differences in mind. To inform campaign design, we surveyed 318 people in Southeast Michigan with a combination of quantitative and qualitative questions designed to assess various axes of PAD (Attention, Attitude, Knowledge, and Relative Interest) and general perceptions of nature. Results were statistically analyzed across gender, education, and age groups and assessed in the context of strategies for mitigation across demographics and axes. We found greater Relative Interest and Attention toward plants in non‐males compared to males and greater Knowledge in the 18–29 age group relative to those 30 and over. Most notably, in a question where participants were asked to construct an ecosystem using abiotic and biotic features, bees were the most commonly selected biotic feature across demographics. We discuss how future plant conservation campaigns can overcome PAD by employing bees specifically as “ambassadors” to increase care for plants and support for policies that protect threatened plant species. This strategy could close demographic gaps in PAD and increase support for plant conservation policies, benefiting society and natural environments.

## 1 Introduction

Even among nature lovers, the degree to which people possess “plant awareness” (Pany et al. 2024; Dünser et al. 2025), i.e., are appreciative of, interested in, and knowledgeable about plants, tends to be surpassed by people's awareness of animals (Wandersee and Schussler 1999). This unconscious bias, known today as “plant awareness disparity” (PAD; Parsley 2020), is likely responsible for a dearth of both scientific work on the conservation of plants relative to animals (Prokop et al. 2022) and policies protecting plant species from detrimental human activity (Margulies et al. 2019). One possible method for addressing PAD is to use public service advertisement campaigns that target the general public, in the vein of the most successful conservation campaigns of the 20th century, such as the United States Forest Service's initiative against human‐caused wildfires represented by Smokey Bear (see Smith 1956) and their campaign against pollution symbolized by Woodsy Owl (see Fuller‐Bennett and Velez 2012).

What should a campaign against PAD look like? Ideally, it would target demographic groups that exhibit the largest degrees of PAD, as it is not evenly distributed. For example, recent research shows that people from rural areas tend to outperform people from urban areas in plant awareness, and women generally exhibit lower PAD than men (Linderwell et al. 2024; Sepúlveda et al. 2025). Additionally, given limitations of information that can be included in a campaign, it would be useful to know which axes of PAD (Attention, Attitude, Knowledge, and Relative Interest, *sensu* Parsley et al. 2022) tend to be strongest across and between demographics.

Therefore, we surveyed to evaluate how the different axes of PAD vary across different demographics (specifically age, gender, and education level) in Southeast Michigan intending to inform future initiatives to improve people's perceptions of plants. The goal of our study was to determine, through quantitative Likert‐scale questions assessing demographic differences in PAD as well as open‐ended qualitative questions asking participants to build their “ideal ecosystem”, to select from a series of animal candidates an effective mascot or mascots for public service campaigns aimed at reducing PAD among the general public.

## 2 Survey Methodology

The survey employed a mixed‐methods approach of Likert‐scale quantitative questions and qualitative questions aimed to integrate objective numerical data with subjective experiences. Prior to commencing subject recruitment, we obtained approval from the University of Michigan's Health Sciences and Behavioral Sciences Institutional Review Board, which ensures that research involving human subjects follows federal and university regulations, poses minimal risk to subjects, and guarantees the protection of subjects' data (IRB number HUM00263691). The survey was deemed to pose “no more than minimal risks” due to the limited interactions with participants, and any possible risks were outweighed by the benefits of understanding PAD in greater depth.

To recruit subjects, we pinned posters with the link to our survey around the University of Michigan (U‐M) campus in Ann Arbor, Michigan, USA, made posts containing the link on various social media websites, and issued a geographically weighted randomized targeted email to 2500 U‐M students. The survey title “Perceptions of Nature” was purposely vague so as to not bias subjects through revealing the focus of the study. Our survey employed five Likert‐scale questions that were used to calculate PAD scores: (1) “When I walk outside, I notice the plants around me”, designed to evaluate Attention; (2) “I have lots of good memories and experiences with plants” and (3) “In my free time, I enjoy going out to spend time in nature”, both of which were designed to evaluate Attitude; (4) “I would rather have plants than animals in my house”, designed to evaluate Interest; and (5) “I perceive grass as living”, designed to evaluate Knowledge. We opted to not use the full version of PAD‐Index for two reasons: (1) this reduced version is briefer, and since we expected most of our survey takers to be undergraduate students, we opted for brevity to increase response rates (see Sarraf and Jony 2026), and (2) we are primarily interested in an exploratory study to inform the design of an awareness campaign for increasing plant awareness. To calculate PAD scores across participants based on answers to these questions, we converted answers on a five‐point scale from “Strongly Disagree” to “Strongly Agree” to numerical scores 1 through 5. We then adapted Parsley's PAD‐Index framework (Parsley et al. 2022) to generate composite scores for each component and total average PAD scores across the four axes.

The second part of our survey aimed to assess subjects' Relative Interest in plants and bees by tasking subjects with the following qualitative question: “Create an ideal ecosystem.” In their answers, respondents could select only two landscape features and two living organisms from the following list of options, each accompanied in parentheses with our justification for inclusion in our survey: tiger (apex predator vertebrate), snake (disliked by many), sunlight (abiotic resource), moss (easily overlooked plant), tree (central forest plant), grass (plant conflated as landscape feature), bees (insect with mixed perceptions among humans), pond (abiotic habitat), deer (prey vertebrate with positive associations), flowers (plant part with positive associations), and butterfly (insect with positive association). If a participant selected more than two living organisms, and one of these three was a plant (tree, grass, or moss), this would indicate low interest because such an answer suggests that the respondent conflated plants with landscape features, meaning that they do not perceive plants as being alive.

To assess how different demographics perceive plants as parts of natural ecosystems, the final part of our survey requested that subjects provide anonymous demographic data (age, optional gender identity, education level). We used this information to examine correlations between these demographics and PAD scores. Specifically, we began with an Exploratory Factor Analysis and calculated mean scores based on factor loadings for the four PAD axes. Then, we employed independent *t*‐tests to compare scores between specific demographic groups (e.g., high vs. low education) and identify any significant (i.e., *p* < 0.05) disparities, and we used both Conover's All‐pairs test (Conover 1971) and chi‐squared tests to evaluate whether differences were significant across several demographic groups. To supplement *t*‐tests, we ran four generalized linear models (GLMs) in which the mean scores for each of the four PAD axes formed the dependent variable and where the independent variables were our three demographic categories (Education + Age + Gender).

## 3 Survey Results

**FIGURE 1:**
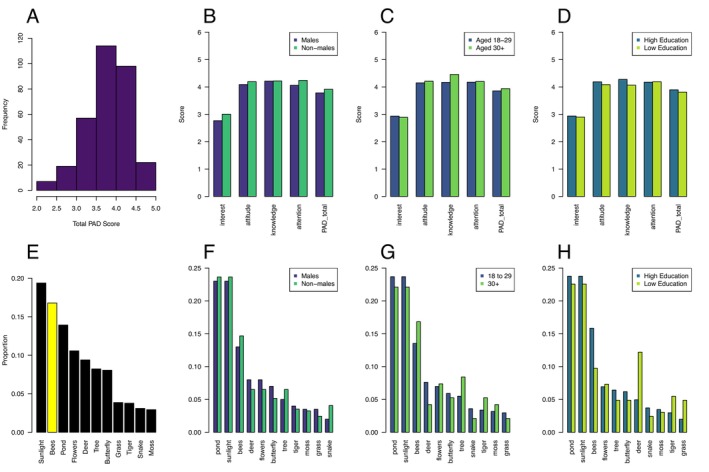
Plots displaying our survey results. (A) Distribution of total PAD scores across all participants. (B) Comparison of mean scores between males and non‐males across the four PAD axes and total PAD scores. (C) Comparison of scores between adults aged 18–29 and adults aged 30+ across the four PAD axes and total PAD scores. (D) Comparison of scores between adults with high education and adults with low education across the four PAD axes and total PAD scores. (E) Proportion of responses to question 1 that included each of the possible ecosystem features (“Bees” is highlighted). (F) Proportion of responses to question 1 broken down by gender. (G) Proportion of responses broken down by age. (H) Proportion of responses to question 1 broken down by level of education.

Our survey had a total of 318 respondents. Most belonged to the 18–29 age range (82.6%), identified as female (60.3%), and reported having some college education (36.3%). The median PAD‐Index across all respondents was 4 (20/25 points on the index); 20 participants scored below 4 and no participants scored below 2, indicating that PAD is relatively low in Southeast Michigan (Figure 1A).

According to *t*‐tests, there were no statistically significant differences in total PAD across gender (*p* = 0.054), age (*p* = 0.36), or education level (*p* = 0.23). However, we did observe some significant differences in PAD‐Index scores when comparing individual axes, specifically: (1) across gender identity, where non‐male (female and non‐binary) participants scored higher on the index for both Relative Interest (Conover's All‐pairs test, *p* = 0.038) and for Attention (*p* = 0.051; see Figure 1B); and (2) across age groups, where the 18–29 age group achieved the highest Knowledge score on the PAD‐Index (*p* = 0.031; Figure 1C). Results from GLM modeling were similar: we found a significant effect only for Gender as a predictor of the mean scores for Relative Interest. All other comparisons between PAD categories and different demographics were not significant, indicating no strong association between PAD scores and most demographic categories we considered.

For the “Create an ideal ecosystem” qualitative question, we found that 66.9% of participants selected bees as one of their living organisms, making bees the second most frequent choice across all options after only sunlight (77.3%). According to a *t*‐test, bees were selected at equal rates in ecosystems designed by participants across both high and low PAD scores (*p* = 0.09), and this finding held true for chi‐square tests comparing the rate of bee selection across gender (*p* = 0.10), age (*p* = 0.14), and education (*p* = 0.11) groups. In other demographic comparisons of responses that included various animals, plants, and abiotic factors, we found no significant differences.

## 4 Bees as Ambassadors for Plant Conservation

**FIGURE 2:**
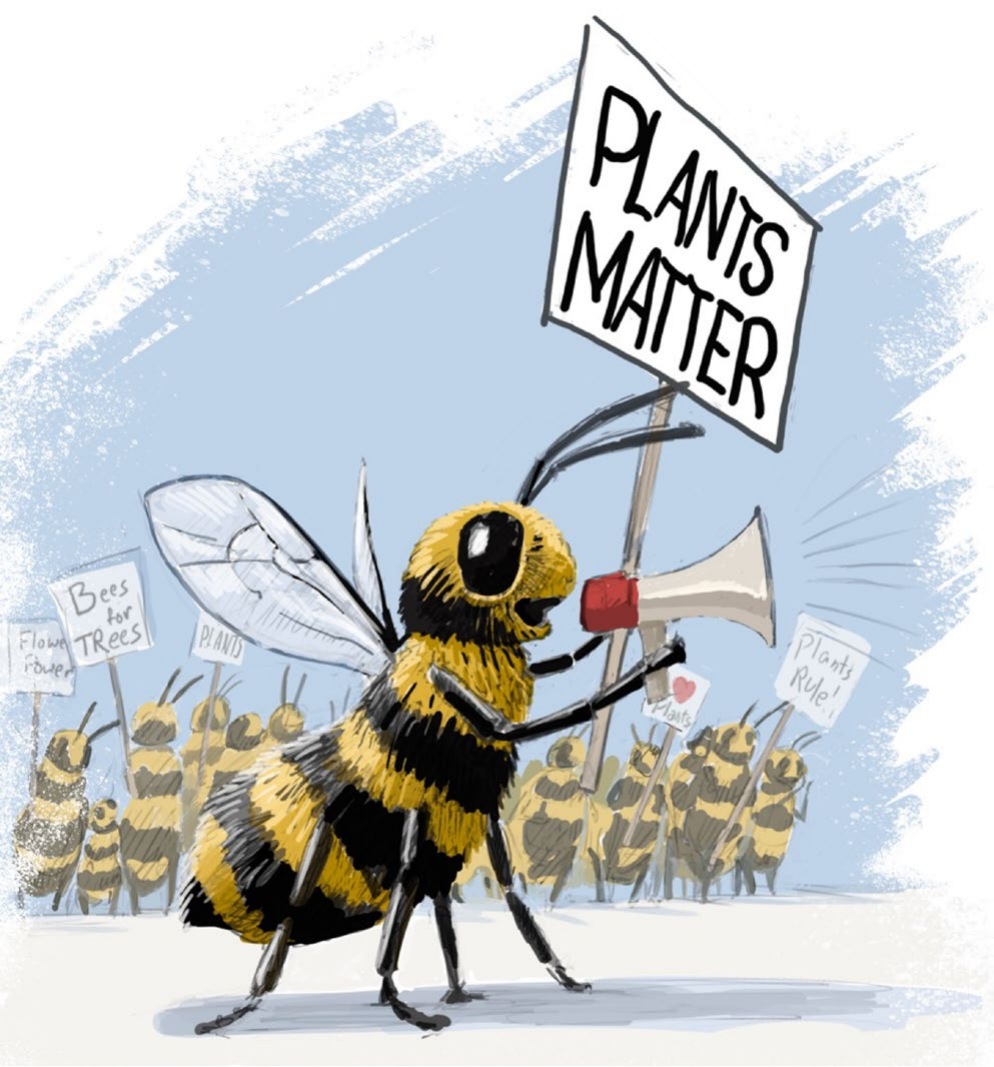
Artist's conception of bees acting as ambassadors for plant conservation. Given our survey findings about perceptions of bees, a similar character might be a good choice as the face of a public service campaign against PAD. Illustration by John Megahan, University of Michigan Museum of Zoology.

Our identification of significantly lower Relative Interest and Attention in males who answered our survey accords with recent research showing that women generally outperform men in plant awareness (Sepúlveda et al. 2025). While our determination that adults aged 18–29 exhibit greater Knowledge relative to adults aged 30+ goes against previous research (Blue et al. 2023; Linderwell et al. 2024), we believe this finding is explained by university students being generally more recently exposed to topics in plant biology than older adults. Our most compelling finding, from qualitative question 1, was that bees were the organism most frequently included in ecosystems designed by participants (Figure 1D). This suggests that bees capture the attention and interest of individuals across demographics, a phenomenon that has been documented in other studies (e.g., Sumner et al. 2018; Burns et al. 2021). Though fear of stinging bees may reduce plant appreciation in some people (Prokop and Fančovičová 2023), our findings suggest that bees may function as “ambassadors” for plant conservation (Figure 2), particularly in bridging demographic gaps, such as the greater incidence of PAD among males relative to non‐males.

Specifically, conservation strategies should capitalize on widespread public interest in bees by explicitly highlighting bee‐plant interdependence, using bees as an entry point to build broader awareness of and support for plant conservation, following the example of campaigns tthat employed animal mascots like Smokey the Bear. Additionally, some of the most popular and captivating nature documentaries (e.g., *Planet Earth*, *The Green Planet*) feature shots of plant–animal interactions to inspire a sense of wonder about the natural world. Therefore, this framing can be used to reduce PAD by increasing interest in plants through popular interest in bees, highlighting plants as essential components of ecosystems rather than background elements.

While the rapid increase in awareness of and empathy for the plight of bees is a major positive development for effective conservation policymaking, it is difficult to imagine a similarly successful campaign on behalf of any plant or plants. While overcoming this “zoocentrism” (Hershey 2002) is still a worthy goal for conservationists, we propose leveraging these major gains to introduce more and more aggressive plant campaigns. Now that a baseline has been established, it is time for conservationists to move to the next step, from the main pollinators of natural flora and agricultural crops (Hristov et al. 2020; Martins et al. 2025) to that which is pollinated. We hope that future studies will systematically quantify the success of plant conservation and campaigns that employ animal ambassadors to determine whether this strategy is effective. In an era of unprecedented and accelerating anthropogenic global change, where native bees are severely threatened by climate change and the negative environmental effects of industrial agriculture (Decourtye et al. 2019), conservationists must be creative in finding effective strategies for increasing awareness and support for powerful environmentalist policies. We only have one planet, after all!

## Funding

The authors have nothing to report.

## Conflicts of Interest

The authors declare no conflicts of interest.

## Data Availability

The data that support the findings of this study and the code used to generate Figure 1 are freely available on Zenodo at https://doi.org/10.5281/zenodo.18843041.
